# West Indian (Caribbean) Punctate Keratopathy in a Puerto Rican Girl

**DOI:** 10.7759/cureus.43467

**Published:** 2023-08-14

**Authors:** Estefania Ramirez Marquez, Alejandra Santiago, Carmen Santos, Armando L Oliver

**Affiliations:** 1 Ophthalmology, University of Puerto Rico School of Medicine, Medical Sciences Campus, San Juan, USA

**Keywords:** west indian punctate keratopathy, uveitis, cornea, puerto rico, wipk, pediatric, caribbean

## Abstract

We report on a case of West Indian (Caribbean) punctate keratopathy (WIPK) in a pediatric patient living in Puerto Rico, USA. A 9-year-old Hispanic girl presented for a routine ophthalmic follow-up. The patient had a history of juvenile idiopathic arthritis and chronic bilateral anterior uveitis. At the presentation, her visual acuity was 20/30 in the right eye and 20/20 in the left eye. An examination of the right eye was remarkable for one round, white subepithelial corneal opacity of approximately 0.1 mm in height by 0.1 mm in width, located slightly lateral to the center of the cornea, which was consistent with WIPK. This case highlights the importance of recognizing WIPK in children who have a history of living on any one or more of the Caribbean islands.

## Introduction

West Indian (Caribbean) punctate keratopathy (WIPK) is characterized by one or more round, white-to-yellow spots in the cornea [1-4]. The lesions have previously been found and described in the corneal subepithelium, specifically in Bowman’s membrane, and may extend toward the stroma [1,2,4,5]. The spots seen in WIPK make it possible to distinguish that disease from others affecting the cornea, as the overlying epithelium remains clear [2].

There have been no ocular complaints or any systemic symptoms associated with WIPK [1-5]. In addition, this condition has not been associated with any other ocular or systemic disease [1-4]. However, WIPK has been found predominantly in adults, of no specific race or ethnicity, who live or have a history of living in the West Indies (Caribbean islands) [1,4]. There are no published reports of WIPK in children of the Greater Antilles (to which group of islands Puerto Rico belongs). We herein present the case of a 9-year-old Hispanic female with a history of juvenile idiopathic arthritis and chronic bilateral anterior uveitis who presented with incidental WIPK.

## Case presentation

A 9-year-old Hispanic female presented for a routine ophthalmic follow-up. Her past medical history was remarkable for her juvenile idiopathic arthritis, diagnosed at 16 months of age, and chronic bilateral anterior uveitis, diagnosed at three years of age. Her medications included methotrexate (0.8 ml) via subcutaneous administration, weekly; prednisolone acetate (1% suspension), every two hours in both eyes (OU); dorzolamide hydrochloride and timolol maleate ophthalmic solution (22.3 mg/6.8 mg per mL), one drop, twice daily OU; and tocilizumab (162 mg/0.9 mL) via subcutaneous administration, every two weeks. Her review of systems, as well as her social and family histories, was otherwise unremarkable at that time.

**Figure 1:**
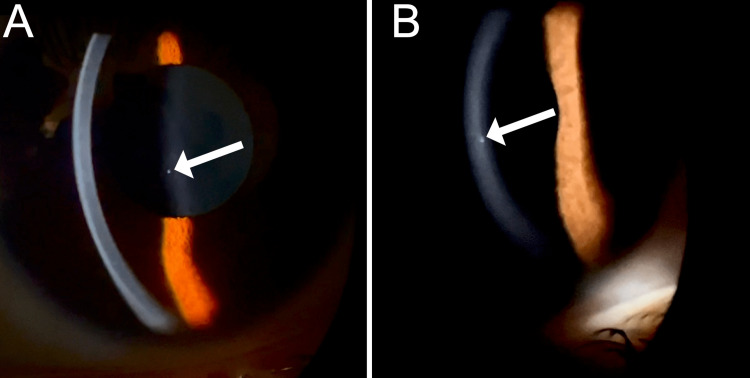
Right eye anterior segment photo with lateral indirect (A) and direct (B) slit-lamp illumination reveals paracentral subepithelial white opacity, measuring 0.1 mm in height by 0.1 mm in width (white arrow). No cells, keratic precipitates, or hypopyon was noted during the examination.

Upon a comprehensive ophthalmic evaluation, her best-corrected visual acuity was 20/30 in the right eye (OD) and 20/20 in the left eye (OS), with a manifest refraction of plano OU. The intraocular pressure was 27 mmHg OD and 12 mmHg OS. The pupils were round and reactive to light, and there was an afferent pupillary defect OD. Her color vision OU, as assessed by the Ishihara color plate test, revealed no defect. Her extraocular movements were within normal limits. A slit-lamp examination (SLE) OD was remarkable for one white, round subepithelial corneal opacity of approximately 0.1 mm in height by 0.1 mm in width, located slightly lateral to the center of the cornea (Figure 1). The rest of the SLE was within normal limits, bilaterally, with no evidence of keratic precipitates, signs of inflammation in the anterior chambers, or vitreous cells in either eye. The patient’s fundus was remarkable for a cup-to-disc ratio of 0.9 OD and unremarkable OS. Gonioscopy was within normal limits OU.

A diagnosis of WIPK was made. The patient returned to the clinic the following month so that her chronic bilateral anterior uveitis could be monitored. Her follow-up SLE showed the WIPK spot to have remained unchanged, and the rest of the ocular examination was unremarkable.

## Discussion

To our knowledge, this is the first pediatric case of WIPK reported in the Greater Antilles, generally, and Puerto Rico (a territory of the US), specifically. The examination of our patient revealed white, round spots located at the level of the corneal subepithelium, which finding is characteristic of WIPK [2,4]. This condition is suspected to be common among people living in the Caribbean; however, the literature about WIPK is very limited [1-5]. The only reference to pediatric cases of WIPK available at this time is the report of an endemic occurrence that happened in Granada in 1993, at which time eight patients aged from 0 to 20 years were incidentally diagnosed with WIPK [3].

Because WIPK is asymptomatic, the spots that identify it tend to be diagnosed by chance, with these diagnoses occurring in patients with unrelated ocular complaints [1-4]. No gene or hereditary pattern has so far been associated with WIPK; furthermore, it has not been linked to any other ocular disease or to anything of a systemic nature [2]. Knowing a patient’s social history is vital to the diagnosis of WIPK, and living or having lived in the Caribbean (i.e., Puerto Rico, as was the case with our patient) is the greatest known risk factor for the disease [1-5]. In the process of diagnosing this condition, the white spots may be distinguished from other types of punctate keratitis, as the corneal epithelium remains normal, and the spots do not change over time or respond to therapy with steroids or antibiotics [3,4]. The patient discussed in this work was being treated with multiple medications, including antimetabolites, immunosuppressants, and anti-inflammatories, none of which affected the appearance of the spot over time.

The etiology of spots of this nature is unknown; however, there are theories that they may be caused by corneal microtrauma, factors associated with agricultural elements, or prolonged exposure to dust from the Sahara Desert, which is carried by the wind currents across the Atlantic Ocean [2-4]. Despite these theories, we cannot rule out the possibility that these spots may be a consequence of various pre-existing ocular pathologies in this patient. The absence of ocular complaints may contribute to the underdiagnosis of and the limited amount of literature available about WIPK. This condition is benign and does not require treatment [1-4]. It is important to quickly recognize WIPK, as its patients may be subjected to unnecessary and/or costly evaluations and treatments. This case highlights the importance of obtaining a thorough social history (from a potential patient), alerts physicians to the possibility that this condition might be affecting pediatric patients, and supports the idea that pharmacotherapy does not alter WIPK spots.

## Conclusions

Our case highlights the significance of recognizing WIPK in children who have a history of living on any one or more of the Caribbean islands. The patient discussed in this work was being treated with multiple medications, including antimetabolites, immunosuppressants, and anti-inflammatories, none of which affected the appearance of the spot over time.
